# Exposure to domestic violence influences pregnant women’s preparedness for childbirth in Nepal: A cross-sectional study

**DOI:** 10.1371/journal.pone.0200234

**Published:** 2018-07-26

**Authors:** Kunta Devi Pun, Poonam Rishal, Jennifer Jean Infanti, Johan Håkon Bjørngaard, Rajendra Koju, Berit Schei, Elisabeth Darj, on behalf of the ADVANCE study group

**Affiliations:** 1 Kathmandu University School of Medical Sciences, Kathmandu University, Dhulikhel, Kavre, Nepal; 2 Department of Public Health and Nursing, Faculty of Medicine and Health Sciences, Norwegian University of Science and Technology, Trondheim, Norway; 3 Central Norway Regional Health Authority, Stjørdal, Norway; 4 Research Centre Brøset, St. Olavs University Hospital, Trondheim, Norway; 5 Department of Obstetrics and Gynecology, St. Olavs University Hospital, Trondheim, Norway; 6 Department of Women’s and Children’s Health, Uppsala University, Uppsala, Sweden; Seoul National University College of Medicine, REPUBLIC OF KOREA

## Abstract

**Objective:**

This study aimed to evaluate if domestic violence affected women’s ability to prepare for childbirth. Birth preparedness and complication readiness (BP/CR) includes saving money, arranging transportation, identifying a skilled birth attendant, a health facility, and a blood donor before childbirth. During data collection, Nepal experienced two earthquakes and therefore it was possible to examine associations between domestic violence, women’s BP/CR and effects of the earthquakes.

**Methods:**

Women who were between 12 and 28 weeks of gestation participated in a descriptive cross-sectional study at a hospital antenatal clinic in Nepal, where they completed a structured questionnaire on sociodemographic characteristics, obstetric history, experiences of domestic violence, and BP/CR. The 5-item Abuse Assessment Screen was used to assess prevalence of domestic violence, and a questionnaire on safe motherhood obtained from Jhpiego was used to assess BP/CR status. The participants self-completed the questionnaire on a tablet computer. Those who reported at least three out of five BP/CR activities were considered prepared for childbirth.

**Results:**

A total of 1011 women participated in the study: 433 pre-earthquakes and 578 post-earthquakes. With respect to BP/CR, 78% had identified a health facility for childbirth and 65% had saved money prior to childbirth. Less than 50% had identified a birth attendant to assist with the delivery, transportation to a health facility, or arranged for a potential blood donor. Prior to the earthquakes, 38% were unprepared; by contrast, almost 62% were not prepared after the earthquakes. A significant association was found between exposure to violence and not being prepared for childbirth (AOR = 2.3, 95% CI: 1.4–3.9). The women with increased odds of not being prepared for childbirth were illiterate (AOR = 9.9, 95% CI:5.7–17), young (AOR = 3.4, 95% CI:1.6–7.2), from the most oppressed social classes (AOR = 3.0, 95% CI:1.2–7.6), were married to illiterate husbands (AOR = 2.5, 95% CI:1.2–5.2), had attended fewer than four antenatal visits (AOR = 2.0, 95% CI: 1.4–2.6), had low incomes (AOR = 1.7, 95% CI:1.1–2.9) or lived in rural settings (AOR = 1.5, 95% confidence interval CI:1.2–2.1).

**Conclusion:**

The paper identifies vulnerable women who require extra care from the health system, and draws attention to the need for interventions to reduce the harmful effects of domestic violence on women’s preparations for childbirth.

## Introduction

Many factors influence pregnancy outcome, with consequences for maternal, newborn and child health. Thaddeus & Maine described already in 1994 ‘The three delay model’, which identifies factors that may inhibit women from accessing antenatal care (ANC) services in limited resource settings. These include delays in seeking care, due to women’s low status and lack of power in health seeking decision-making and lack of awareness of danger signs among pregnant women and their close relatives. The model also identifies groups of factors resulting in delays in arrival at a health facility, such as long distances, lack of arrangements and finances for transportation and poor infrastructure. Furthermore, delays in provision of adequate care at the facility, availability of skilled care providers and equipment [1]. Domestic violence (DV) and men’s controlling behaviors are also factors that influence women’s ability to make decisions concerning their health, which could improve their pregnancy outcomes [2].

Antenatal care (ANC) from a skilled provider may be able to reduce the risks of maternal and infant mortality and morbidity during pregnancy and childbirth [3]. The maternal mortality ratio (MMR) is a strong indicator of the overall effectiveness of a national health system. In Nepal, the MMR in 2015 was 258 deaths per 100,000 live births [4], which was significantly higher than elsewhere in the world, and far below the Sustainable Development Goals target of not more than 70 deaths per 100,000 live births by the year 2030 [5]. Pregnant women meet the same health care worker, such as midwives multiple times, which creates an opportunity to build trusting relationships and it has been reported that repeated interviews and screening leads to higher rates of identification of DV [6]. A Swedish study conducted in antenatal settings, examined midwives’ experiences of women’s responses to their questions about men’s violence [7]. The midwives described their roles as raising awareness of the problem, reducing the shame associated with being a victim of abuse, giving information and emotional support [7]. The same researchers also examined the women’s experiences of being asked about violence and they found that most pregnant women (80%) were not opposed to midwives asking about their exposure to violence as part of routine identification of risk factors carried out for every pregnancy [8]. Such disclosures of DV may provide opportunities for health care workers to give information and advice on safety behaviors and how to access safe houses, counselling and other support services, and to make referrals. There is scant evidence of improved rates of midwives’ detection of DV in low-income countries. However, a recent Sri Lankan study shows that improvements were made in midwives’ detection of DV and the provision of assistance and knowledge following a training program that targeted intimate partner violence and lowering the barriers to discussing the issue [9]. Evidence from high-income countries also suggests that the health outcomes and safety of women living with DV can be improved through interventions to identify survivors and introduce context-specific, safety planning methods to women [10].

Focused antenatal care (FANC)—an approach launched by the World Health Organization (WHO) in 2002 [11]—targets all pregnant women who are at risk of developing complications. The FANC approach aims to give holistic and individualized care to all pregnant women in order to maintain normal progress in pregnancy. FANC ensures timely guidance and advice on birth preparedness and complication readiness (BP/CR). The concept of BP/CR addresses the delays described in Thaddeus & Maine’s model to seeking, reaching, and receiving health care [1], which result in numerous maternal deaths worldwide [3].

Nepal’s Ministry of Health and Population (MoHP) endorses the FANC approach, and recommends that all pregnant women receive basic monitoring and care during pregnancy, and that information and screening for potential complications be provided to them routinely during all ANC visits. The MoHP has implemented a package of birth preparedness interventions as part of Nepal’s Safe Motherhood Plan (2002–2017). The package outlines a number of actions that pregnant women and their families should take to prevent delays in accessing childbirth services. Such proactive planning is known to save women’s lives, especially in rural locations [12]. Worldwide, reports of preparedness for childbirth vary from 12–65% in studies conducted in Ethiopia, Uganda, Nigeria and Tanzania [13–18], to 35–62% [19–23] in India, and 32–65% [24–27] in Nepal.

A hypothesis for this study was that woman’s ability to fulfill the criteria of BP/CR can be negatively influenced by experience of DV. DV is defined as any form of physical, mental, sexual, or economic harm, including acts of reprimand or emotional harm perpetrated by one person against another with whom he or she has a familial relationship [28]. One of the main characteristics of DV is controlling behavior, which can include control over women’s resources when preparing for childbirth and access to ANC. Another characteristic is the significant association between intimate partner violence and numbers of pregnancy-associated suicides and homicides [29]. Furthermore, a woman’s experience of DV during pregnancy can have detrimental effects for her unborn child [30]. A multicountry study conducted by the World Health Organization (WHO) revealed that the prevalence of abuse during pregnancy ranged from 1% (Japan) to 28% (Peru) [31]. In Campbell, Garcia-Moreno and Sharps’s review of studies conducted in developing countries [32], the percentage of physical abuse during pregnancy is reported as ranging from 3.5% (China) to 31.7% (Egypt).

According to the Nepal Demographic Health Survey (NDHS) made in 2011, 22% of women in the age range 15–49 years had experienced physical violence at least once since the age of 15 years, and 12% of women had experienced sexual violence at least once in their lifetime [12]. One-third of the women who experienced any type of DV reported their spouse as the perpetrator of the violence. In the same survey, estimates of violence during pregnancy ranged from 2% among women with higher education levels to 10% among women who were divorced, separated, or widowed [12]. The NDHS also found that 50% of pregnant Nepalese women attended all four recommended ANC visits [12]. It is clear from the survey that ANC serve as a common point of contact between pregnant women and representatives of the health care system in Nepal. Hence, pregnant women’s attendance at ANCs are valuable opportunities for health care workers to identify and respond to women living with DV, in order to prevent or reduce adverse maternal and neonatal health outcomes associated with such violence. No prior studies of the influence of DV on BP/CR have been conducted in Nepal. Two major earthquakes occurred during the process of data collection. According to studies from China and Japan, women are at increased risk of DV after natural disasters [33, 34]. Since there is scant evidence of how large-scale disasters such as earthquakes may affect women’s BP/CR, we chose to examine associations between women’s BP/CR status before and after the two earthquakes in Nepal. Hence, the aim of this study was to determine if exposure of DV affects women’s BP/CR and if the cumulative effect of multiple obstacles of barriers interacted.

## Materials and methods

### Study setting

The study was conducted in the antenatal clinic of Dhulikhel Hospital-Kathmandu University Hospital (DH-KUH), which is located in Dhulikhel Municipality, Kavre District, 30 kilometres east of the capital city of Kathmandu. Dhulikhel’s population of 14,283 live in a total of 3279 households [35]. However, the hospital provides services to approximately 1.9 million people from more than 50 out of 75 districts in the country [36]. The antenatal clinic at DH-KUH is run by midwives for normal pregnancies; high risk cases are referred to obstetricians. In 2015, the hospital received women for a total of 14,612 antenatal visits and there were 2865 childbirths [37].

### Study design and population

From November 2014 to November 2015, a cross-sectional descriptive study was conducted at the hospital ANC clinic, utilizing a self-completed questionnaire. Women visiting the ANC clinic during this period, who met the study’s inclusion criteria and were between 12 and 28 gestational weeks, were invited to participate in the study. Women who were visually, orally, audibly and mentally disabled, severely ill, or could not speak or understand Nepali language, were excluded from the study.

### Questionnaire

A questionnaire was designed and administered utilizing a color-coded audio computer-assisted self-interview (C-ACASI) in a tablet computer. The participating women received a tablet and headset, and could listen to the interview questions through the headset while also reading the same questions on the tablet. This allowed the inclusion of both literate and illiterate women in the study. The audio-recording and text were in Nepali language. The women answered the questions by touching color-coded buttons on the screen, indicating “yes” or “no.”

A five-item questionnaire, the Abuse Assessment Screen (AAS), was used to identify DV [38]. The order of one of the AAS questions was modified in our study. Rather than starting the questionnaire with the original first question in the AAS—“Have you ever been emotionally or physically abused by someone important in the family to you?”—we chose to begin with the fifth question, “Are you afraid of anyone within your family?” This modification was based on the assumption that the changed order facilitated a broader and gentler introduction to the questionnaire, rather than immediately beginning with the word “abuse.” A questionnaire for birth preparedness and complication readiness, developed by the Johns Hopkins Program for International Education in Gynecology and Obstetrics (Jhpiego) for its safe motherhood and neonatal health program, was used to assess the status of BP/CR among the pregnant women [3, 38]. Sociodemographic information and obstetric history were also included in the questionnaire.

The complete questionnaire was translated from English to Nepali separately by two professional translators. The first and second authors, both Nepalese, cross-checked the wording in the translated questionnaire before starting data collection and simplified a few words. Then the questionnaire was recorded and the audio file transferred to the C-ACASI platform. The same technique has been used in India, showing both reliability and feasibility for sensitive research topics [39, 40]. To our knowledge, this is the first time this questionnaire tool has been used in Nepal. The C-ACASI was pre-tested among pregnant women at Kathmandu Medical College and Teaching Hospital (KMC) and no changes were needed for use in the study setting in Dhulikhel.

### Sample size

**Fig 1:**
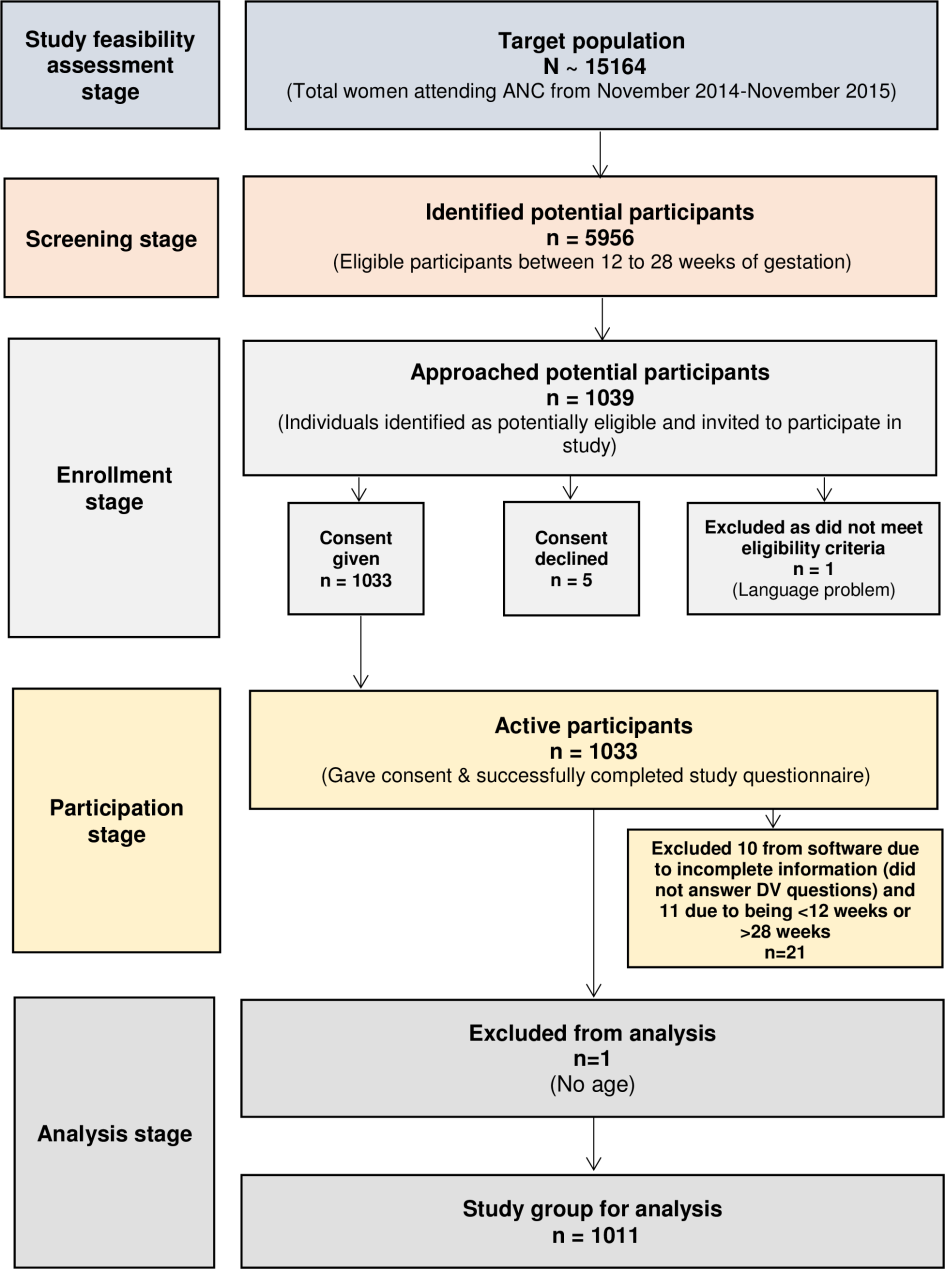
Flow chart of inclusion in the study population.

Over the course of the study, nearly 6000 women were potentially eligible for the study (Fig 1), but due to staff and software resources, it was only possible to approach 1039 of the eligible women. Of them,1033 consented to participate. Ten women were excluded later as they chose to opt out before answering the questions on violence; 11 were excluded as it became apparent they did not meet the inclusion criteria (for example, they were outside the gestational period of 12–28 weeks); and one was excluded due to missing information. Therefore, data was analyzed from 1011 women, or 97.3% of the women completed the questionnaire (Fig 1). The women were only included once in the study in the same pregnancy.

### Data collection

Upon arrival at the antenatal clinic at DH-KUH for a routine visit, all women met a receptionist-midwife. Women with high risk factors were directly referred to an obstetrician. From each woman’s ANC card, the midwife was able to determine her eligibility for the study. Eligible women were informed about an ongoing study titled “Women’s reproductive health.” Each woman was asked if she would like to receive further details about the study and potentially participate. If she agreed, she was accompanied to a separate research room in the clinic where the first author or a research assistant provided her with further information about the study as part of the consent procedure. If the woman declined, she continued with her ANC appointment as normal. However, if she gave consent to participate in the study, she was shown how to use the tablet computer to answer the study questionnaire and assigned a study number on her ANC card. Responding to the questionnaire required on average 15–30 minutes. Upon completion, the woman was accompanied by the data collector to the regular clinic room for her ANC appointment.

### Variables

#### Birth preparedness and complication readiness

The concept of BP/CR comprises five activities: 1) saving money for birth-related and emergency expenses; 2) identifying the location of the closest health care facility; 3) identifying a skilled birth attendant; 4) identifying available transport; and 5) identifying blood donors in case of an emergency [3]. The response alternatives for the questions on BP/CR were “yes” or “no” in the tablet computer. In our definition, a woman was considered to be “*Prepared*” for childbirth and ready for potential complications, if she reported having undertaken at least three of the five basic BP/CR activities. Those positively responding to two or fewer activities were considered “*Not prepared*.” This scoring system has been used previously in other studies [17, 18, 41].

#### Domestic violence

The five-item AAS was used to detect DV experienced by pregnant women. This originally American screening tool has been tested internationally with low-income populations and uninsured women, in Brazil and Sri Lanka [42]. Women in our study were classified as having experienced “*Any DV*” if they reported the experience of fear of and/or violence from someone in the family. Women classified as having experienced “*Fear only*” are those who gave a positive answer to the question on being afraid of someone in the family but whom answered “*No*” to experiencing violence. Women classified as reporting “*Violence*” were those with a positive response to any of the questions on physical, emotional and sexual violence, regardless of reporting fear of someone in the family or not. Women reporting having been afraid of someone in the family, independent of their response to the other abuse questions, were classified as having “*Fear*.” Women classified as never experiencing neither fear nor violence were considered to be not exposed to domestic violence: “*No domestic violence*.” Women who had experienced violence were also asked about the time point in which the violence occurred: ever, in the last year, and/or during the current pregnancy.

#### Sociodemographic characteristics, obstetric history and earthquake status

The interview included questions about age, education, the income of the woman and her husband, a woman’s autonomy to use her own income, geographic setting, and family structure. Family structure was categorized as either a nuclear family, where the husband, wife and children are living separately from their in-laws, or an extended family, where a woman is living with her husband, children, and in-laws and possibly other members of their families. The variables “Women’s own income” and “Autonomy to use income” were computed into three categories: “*No income no autonomy*,” “*Income but no autonomy*” and “*Income and autonomy*.” The women were also categorized based on caste/ethnicity, which was self-reported and in their identification cards. Members of Dalit communities belong to the most oppressed social class in Nepal; “Disadvantaged Janajati” are indigenous groups with little or no social mobility; “Advantaged Janajati” are indigenous groups with opportunity and access to social mobility, and “Upper castes” are traditionally the most privileged groups in the social hierarchy.

The obstetric variables collected included parity, number of antenatal visits, gestational age and pregnancy complications. Of these, “Parity” was computed into three categories: “*First pregnancy*,” “*Previous pregnancy without complication*” and “*Previous pregnancy with complication*.”

An earthquake variable was also included following the two major earthquakes that affected Nepal during data collection, on 25 April and 12 May in 2015. The earthquakes caused extensive damage with reports of nearly 9000 deaths and 23,000 injuries [43]. Inclusion of participants into the study resumed three weeks after the last earthquake. Adding the pre- and post-earthquakes variables granted an opportunity to investigate the consequences of the natural disaster on women’s BP/CR.

### Data analysis

The collected data were analyzed using SPSS version 22. Descriptive analysis was performed to assess the relationships between women’s BP/CR status and their sociodemographic, obstetric, earthquake status, and DV characteristics and experiences. The five BP/CR activities, along with a composite score for BP/CR, were compared with experiences of DV and with pre- or post-earthquake status. The prevalence rates for different kinds of domestic violence were compared with the BP/CR statuses.

In this study, complete case information was obtained from 954 women after adjusting for the variables of women’s age, education and parity. With this population, logistic regression analysis was used to identify the factors associated with BP/CR, by calculating crudes odds ratios and adjusted odds ratios, with 95% confidence intervals (CI).

### Ethical considerations

Permission for the study was granted by the Regional Committee for Medical and Health Research Ethics of Central Norway (REK 2014/613) in Norway, the Nepal Health Research Council (NHRC 81/2014), and the Kathmandu University Institutional Review Committee (KUIRC 55/14).

The objective of the study, the principle and practical meaning of “voluntary participation’, and possibilities to opt out of the study at any time without giving a reason, were thoroughly explained to the participants before they completed the questionnaires. Verbal consent was approved by the ethics committees and chosen over written consent, due to low literacy rates in the study area. In total, 40% of women and 14% of men have not received a formal education in Nepal [12]. The verbal consent of participants was registered by the data collector before participation started. The participants completed the questionnaires in a private room to ensure their privacy and confidentiality. Additionally, to ensure safety, study participants received an information brochure called “Safe motherhood,” produced by the government of Nepal. They also received an anonymous-looking business card with a common female forename and a telephone number printed on it. The card was the contact number for the female psychosocial counsellor employed at the One-stop Crisis Management Centre (OCMC) for victims of gender-based violence, located at Dhulikhel Hospital. These strategies were designed to assist women in potentially justifying why their visits with the midwives took longer than possibly anticipated for anyone accompanying them to the ANC clinic, and in the case that the women did not want to disclose information about answering questions on DV as part of the questionnaire. All study participants were provided the option of private counselling if needed following the interview, but none availed of this.

The mean age of marriage for women in Nepal is 17.4 years [12]. Thus, in order to include all fertile women in the study, we included women under age 18, following the age categories in the NDHS. As DV is a sensitive topic and may be hidden from or perpetrated by close relatives, parental consent was not sought on behalf of these women. This was approved by the ethical committees.

## Results

Among the 1011 study participants, 45% resided in urban areas and the remaining in rural settings. Ninety-nine percent were married and in a first marriage, and 96.5% were their husband’s only wife. The majority, 61.3%, lived in extended families. The women had a mean age of 24.4 years (SD 4.0) and their husbands were a few years older, 27.6 years (SD 4.6).

As shown in Table 1, slightly more than three-fourths of women, 76.3%, were not exposed to DV, 14% reported they were afraid of someone in the family but not exposed to violence, and 9.7% had experience of both DV and fear. Among the women who were prepared for childbirth according to our definition, 83.3% had no experience of violence, compared with the women who were not prepared for childbirth, of whom 69.4% had no experience of DV. Among the women who were not prepared, most were from rural areas, had no income of their own nor autonomy to use the available means in the family. Women who were pregnant for the first time and in the second trimester did not prepare for childbirth as consistently as women with previous pregnancy experience. The proportion of women who did not prepare for childbirth increased after the earthquakes. In total, 61.7% of the women were not prepared post-earthquakes, compared with 38.3% before the earthquakes.

**Table 1:** Distribution of domestic violence, earthquake status, sociodemographic and obstetric characteristics in relation to birth preparedness and complication readiness among pregnant women in Nepal (N = 1011).

Characteristics	Total		BP/CR^a^				
			Prepared		Not prepared		
	N = 1011		n = 502		n = 509		
	n	(%)	n	(%)	n		(%)
***Domestic violence***							
No domestic violence	771	(76.3)	418	(83.3)	353		(69.4)
Fear only^b^	142	(14.0)	53	(10.6)	89		(17.5)
Violence^c^	98	(9.7)	31	(6.2)	67		(13.2)
***Earthquakes***							
Before	433	(42.8)	238	(47.4)	195		(38.3)
After	578	(57.2)	264	(52.6)	314		(61.7)
***Age of women in years (16–45) [mean(SD)]***	24.4(4.0)		24.9 (3.9)		23.8 (4.1)		
***Age of husband in years (14–52) [mean(SD)]***	27.6(4.6)		28.2(4.4)		26.9(4.8)		
***Geographic setting***							
Rural	556	(55.0)	242	(48.2)	314		(61.7)
Urban	455	(45.0)	260	(51.8)	195		(38.3)
***Caste and ethnicity***							
Dalit^d^	31	(3.1)	7	(1.4)	24		(4.7)
Disadvantaged Janajati^e^	250	(24.7)	86	(17.1)	164		(32.2)
Advantaged Janajati^f^	261	(25.8)	149	(29.7)	112		(22.0)
Upper castes^g^	469	(46.4)	260	(51.8)	209		(41.1)
***Education of women (n***^h^ ***= 1008)***							
No education (not able to read)	111	(11.0)	23	(4.6)	88		(17.4)
Primary education (grade 1–5)	185	(18.4)	60	(12.0)	125		(24.7)
Secondary education (grade 6–10)	232	(23.0)	101	(20.1)	131		(25.9)
Higher secondary and above (grade 11+)	480	(47.6)	318	(63.3)	162		(32.0)
***Women's own income and autonomy to use***							
No income no autonomy	766	(75.8)	329	(65.5)	437		(85.9)
Income but no autonomy	71	(7.0)	42	(8.4)	29		(5.7)
Income and autonomy	174	(17.2)	131	(26.1)	43		(8.4)
***Married***							
Yes	1002	(99.1)	500	(99.6)	502		98.6)
No	9	(0.9)	2	(0.4)	7		(1.4)
***First marriage (n = 1002)***							
Yes	992	(99.0)	498	(99.6)	494		98.4)
No	10	(1.0)	2	(0.4)	8		(1.6)
***Husband only wife (n = 1002)***							
Yes	967	96.5)	494	(98.8)	473		(94.2)
No	35	(3.5)	6	(1.2)	29		(5.8)
***Family structure (n = 990)***							
Nuclear	383	(38.7)	192	(38.9)	191		(38.5)
Extended	607	(61.3)	302	(61.1)	305		61.5)
***Parity (n = 957)***							
First pregnancy	503	(52.6)	235	(49.2)	268		(55.9)
Previous pregnancy without complication	387	(40.4)	202	(42.3)	185		(38.6)
Previous pregnancy with complication	67	(7.0)	41	(8.6)	26		(5.4)
***Gestational age (n = 1003)***							
12–24 weeks	814	(81.2)	403	(80.9)	411		(81.4)
25–28 weeks	189	(18.8)	95	(19.1)	94		(18.6)
***Antenatal visits***							
< 4	660	(65.3)	287	(57.2)	373		(73.3)
≥ 4	351	(34.7)	215	(42.8)	136		(26.7)

^a^BP/CR = Birth preparedness and complication readiness

^b^Fear only = Fear but no violence

^c^Violence = Violence regardless of fear

^d^Dalit = The most oppressed social class

^e^Disadvantaged Janajati = Indigenous groups with little or no social mobility

^f^Advantaged Janajati = Indigenous groups with opportunity and access to social mobility

^g^Upper castes = Traditionally, the most privileged groups in the social hierarchy

^h^n varies according to missing information

As shown in Table 2, half of the women in our study were not prepared for childbirth. Slightly more than three-quarters, 77.6%, of the women had identified a health facility for the delivery and/or for obstetric emergencies. More than half had saved money, 64.5%, for the anticipated costs of delivery and emergency if needed. Half of the participants had identified a skilled birth attendant. Less than half of the women arranged transportation, and 20.1% had identified a potential blood donor. However, women with experience of DV were significantly less prepared than those without DV in all of the five activities (p-value < 0.001 in the composite score). Following the earthquakes, women were also significantly less prepared, with the most pronounced decline being in arrangements for transportation (p-value < 0.001) and identification of a skilled birth attendant (p-value 0.01).

**Table 2:** Birth preparedness and complication readiness status of pregnant women in relation to experience of domestic violence and earthquakes in Nepal (N = 1011).

Birth preparedness and complication readiness	Total		Any DV^a^				p-value	Earthquake				p-value
			Yes		No			Before		After		
	N = 1011		n = 240		n = 771			n = 433		n = 578		
	n	(%)	n	(%)	N	(%)		n	(%)	n	(%)	
Identified health facility for childbirth/emergency	785	(77.6)	161	(67.1)	624	(80.9)	<0.001	340	(78.5)	445	(77.0)	0.563
Saved money for possible emergency	652	(64.5)	130	(54.2)	522	(67.7)	<0.001	295	(68.1)	357	(61.8)	0.036
Identified skilled birth attendant for childbirth	504	(49.9)	98	(40.8)	406	(53.7)	0.001	236	(54.5)	268	(46.4)	0.01
Arranged for transport	384	(38.0)	72	(30.0)	312	(40.5)	0.004	193	(44.6)	191	(33.0)	<0.001
Identified potential blood donor	203	(20.1)	32	(13.3)	171	(22.2)	0.003	97	(22.4)	106	(18.3)	0.111
Prepared (Composite score ≥ 3)	502	(49.7)	84	(35.5)	418	(54.2)	<0.001	314	(54.3)	195	(45.0)	0.003

Multiple responses possible

^a^Any DV = Any domestic violence (fear and/or violence or both)

Approximately one-quarter of the women, 23.7%, reported experiences of any DV. A substantially higher percentage of the women who were not prepared were among the women exposed to DV. A total of 203 women, 20.1%, reported being afraid of someone in the family at the time of completing the questionnaire, while 6.1% reported having experienced emotional and physical abuse combined, 4% physical abuse only, 2.3% physical abuse during the current pregnancy, and 1.6% had been sexually abused (Table 3).

**Table 3:** Prevalence of domestic violence and birth preparedness and complication readiness among pregnant women in Nepal (N = 1011).

Domestic violence		Total		BP/CR^a^				p-value
				Prepared		Not prepared		
		N = 1011		n = 502		n = 509		
		n	(%)	N	(%)	n	(%)	
**Any DV**^b^		240	(23.7)	84	(16.7)	156	(30.6)	<0.001
	Fear only^c^	142	(14.0)	53	(10.6)	89	(17.5)	
	Violence^d^	98	(9.7)	31	(6.2)	67	(13.2)	
**Ever**								
	Fear^e^	203	(20.1)	73	(14.5)	130	(25.5)	<0.001
	Emotional or physical abuse	62	(6.1)	24	(4.8)	38	(7.5)	0.075
**Previous year**								
	Physical abuse	40	(4.0)	12	(2.4)	28	(5.5)	0.018
	Sexual abuse	16	(1.6)	5	(1.0)	11	(2.2)	0.218
**Since pregnant**								
	Physical abuse	23	(2.3)	6	(1.2)	17	(3.3)	0.038

Multiple responses possible

^a^BP/CR = Birth preparedness and complication readiness

^b^Any DV = Any domestic violence (fear and/or violence or both)

^c^Fear only = Fear but no violence

^d^Violence = Violence regardless of fear

^e^Fear = Afraid of anyone in the family

In the binary logistic regression analysis, for the 509 of 1011 women who were not prepared for childbirth there was a statistically significant association between exposure to violence and not being prepared (AOR = 2.3, 95% CI:1.4–3.9) (Table 4).

**Table 4:** Associated risk factors for birth preparedness and complication readiness among pregnant women with complete case information (N = 954) in Nepal.

Characteristics	Not prepared^d^		BP/CR^a^			
	n	(%)	Crude OR^b^ (95%CI^c^)		Adjusted OR (95% CI)	
**Domestic violence**						
No domestic violence	331	(69.5)	1(Ref)		1(Ref)	
Fear only	84	(17.6)	2.1	(1.5–3.1)	1.8	(1.2–2.6)
Violence^e^	61	(12.8)	2.6	(1.6–4.1)	2.3	(1.4–3.9)
**Earthquake status**						
Before earthquake	183	(38.4)	1(Ref)		1(Ref)	
After earthquake	293	(61.6)	1.5	(1.0–1.9)	1.4	(1.0–1.8)
**Sociodemograpbhic**						
***Age of women***						
15–19	52	(10.9)	4.4	(2.3–8.5)	3.4	(1.6–7.2)
20–24	234	(49.2)	1.6	(1.0–2.4)	1.8	(1.1–2.9)
25–29	144	(30.3)	1.1	(0.7–1.8)	1.5	(0.9–2.4)
≥ 30	46	(9.7)	1(Ref)		1(Ref)	
***Age of husband***						
≤ 24	150	(32.1)	2.4	(1.4–4.0)	1.7	(0.8–3.4)
25–29	183	(39.2)	1.1	(0.7–1.8)	1.1	(0.6–2.1)
30–34	99	(21.2)	1.2	(0.7–1.9)	1.4	(0.8–2.6)
≥ 35	35	(7.5)	1(Ref)		1(Ref)	
***Family structure***						
Nuclear	176	(38.0)	1(Ref)		1(Ref)	
Extended	287	(62.0)	1.0	(0.8–1.3)	1.2	(0.9–1.6)
***Geographical setting***						
Rural	293	(61.6)	1.7	(1.3–2.1)	1.5	(1.2–2.1)
Urban	183	(38.4)	1(Ref)		1(Ref)	
***Caste and ethnicity***						
Dalit^f^	22	(4.6)	3.9	(1.6–9.3)	3.0	(1.2–7.6)
Disadvantaged Janajati^g^	149	(31.3)	2.3	(1.6–3.1)	1.4	(1.0–2.1)
Advantaged Janajati^h^	105	(22.1)	0.9	(0.7–1.3)	0.9	(0.6–1.2)
Upper castes^i^	200	(42.0)	1(Ref)		1(Ref)	
***Education of women***						
No education (not able to read)	81	(17.0)	8.1	(4.8–13.8)	9.9	(5.7–17.1)
Primary education (grade 1–5)	121	(25.4)	4.2	(2.9–6.1)	4.9	(3.3–7.2)
Secondary education (grade 6–10)	123	(25.8)	2.5	(1.8–3.5)	2.7	(1.9–3.8)
Higher secondary and above (grade 11+)	151	(31.7)	1(Ref)		1(Ref)	
***Education of husbands***						
No education (not able to read)	39	(8.4)	5.3	(2.8–10.2)	2.5	(1.2–5.2)
Primary education (grade 1–5)	130	(27.8)	4.2	(2.9–6.1)	2.2	(1.4–3.4)
Secondary education (grade 6–10)	132	(28.3)	2.1	(1.5–2.8)	1.4	(1.0–2.0)
Higher secondary and above (grade 11+)	166	(35.5)	1(Ref)		1(Ref)	
***Income of husbands***						
Low < 8000 NRS^j^ per month	59	(18.4)	2.7	(1.7–4.3)	1.7	(1.1–2.9)
Middle 800016,000 NRS per month	108	(33.8)	1.3	(1.0–1.8)	1.2	(0.8–1.7)
High > 16,000 NRS per month	153	(47.8)	1(Ref)		1(Ref)	
***Women's own income and autonomy to use***						
No income no autonomy	408	(85.7)	1(Ref)		1(Ref)	
Income but no autonomy	28	(5.9)	0.6	(0.4–1.0)	0.7	(0.4–1.2)
Income and autonomy	40	(8.4)	0.3	(0.2–0.4)	0.4	(0.2–0.6)
**Obstetric history**						
***Parity***						
First pregnancy	266	(55.9)	1(Ref)		1(Ref)	
Previous pregnancy without complication	184	(38.7)	0.8	(0.6–1.1)	0.7	(0.5–0.9)
Previous pregnancy with complication	26	(5.5)	0.6	(0.3–0.9)	0.4	(0.2–0.8)
***Antenatal visits***						
< 4	350	(73.5)	2.0	(1.5–2.6)	2.0	(1.4–2.6)
≥ 4	126	(26.5)	1(Ref)		1(Ref)	

Adjusted for women’s age, education and parity

^a^BP/CR = Birth preparedness and complication readiness

^b^OR = Odds ratio

^c^95% CI = Confidence interval at 95%

^d^Not prepared = Those women who had made ≤2 preparation for childbirth out of 5 BP/CR activities

^e^Violence = Violence regardless of fear

^f^Dalit = The most oppressed social class

^g^Disadvantaged Janajati = Indigenous groups with little or no social mobility

^h^Advantaged Janajati = Indigenous groups with opportunity and access to social mobility

^i^Upper castes = Traditionally, the most privileged groups in the social hierarchy

^j^NRS 1000 = USD 9.40 (February 2017)

Further, being of young age, living in rural areas, being of Dalit or other disadvantaged ethnic group status, having no or limited formal education, having a husband with low or no education and low income, and attending less than four antenatal visits, were all positively associated with not being prepared for childbirth. However, there was no substantial association between age of husband or family structure and BP/CR. Two additional predictors for being prepared were having personal income and the autonomy to use it (AOR = 0.4, 95% CI:0.2–0.6), and having previous pregnancies with (AOR = 0.7, 95% CI:0.5–0.9) or without complication (AOR = 0.4 95% CI:0.2–0.8). We did not find support for statistical interaction between being exposed to domestic violence and earthquake status and the odds of not being prepared (p-value statistical interaction ≥0.05). Compared with no DV, women reporting fear only had a 2.5 times increased crude odds of not being prepared (OR = 2.5, 95% CI:1.4–4.4) prior to the earthquake. The corresponding odds ratio after the earthquake was 1.9 (95% CI:1.1–3.3). Compared with no DV, women exposed to violence had a 2.4 times increased crude odds of not being prepared (OR = 2.4, 95% CI:1.3–4.5) prior to the earthquake. The corresponding odds ratio after the earthquake was 3.2 (95% CI:1.5–6.7).

## Discussion

Several factors will influence a woman’s ability to prepare for childbirth. In this study, we focused on exposure to DV, as we identified a gap in information on this matter in developing countries. We found that almost one-quarter of the participants had experience of some kind of DV and they were twice as likely not to be prepared for childbirth compared with those who reported no experience of DV. Vulnerable women in poor social circumstances were more likely to not being prepared for childbirth. Whereas, personal income and autonomy to use it, were predictors for preparing for childbirth. These factors reflects the influence of women’s social and economic position on their ability to preparing for childbirth in a patriarchal society.

Within the health system, ANC provides services that most women receive during their lifetime, also in Nepal. An ongoing relationship with an ANC provider may facilitate the development of the trust needed for a woman to disclose the occurrence of DV and to accept information about safety planning and BP/CR. Much of the evidence on effective interventions to address DV in ANC contexts has come from high-income countries. Without similar knowledge from low-income settings, interventions and guidelines in the health sector may not accurately reflect the realities for millions of women. Furthermore, many women in low-income settings such as Nepal are unable to leave violent relationships, and therefore it is critical to mitigate the harm done to them.

### Domestic violence

Our findings, that women with experiences of DV did not prepare for childbirth as other women, are similar to those from a community-based study of women with a child under the age of two years in India [44], which demonstrated significant associations between violence and poor birth preparedness. This highlights the importance to public health of ANC providers routinely asking pregnant women if they are living in violent situations. In addition to ANC and other health care providers, communities can play a role in supporting pregnant women’s preparations for childbirth, especially those living with DV. This was expressed in a recent study in the same community as we studied, where men and women of all ages and family roles gave suggestions to the researchers for potential improvements that would benefit women living with DV during pregnancy[2]. For example, they suggested community awareness-raising programs about the health effects of DV, and engaging extended family members to address DV and help women to attend antenatal check-ups [2].

### Birth preparedness and complication readiness

According to our definition of BP/CR, half of the women were not prepared for childbirth. This is higher than found in a number of community-based studies of women who had experienced childbirth in Nepal [24], India [19, 23], and Ethiopia and Tanzania [13, 14, 45, 46] in recent years. The difference in findings may be due to differences in the study areas or to the implementation of different health programs by government or non-governmental organizations in those areas [27]. Hence, our results may reflect selection bias, since our study was hospital based, while the other studies were not. It is reasonable to assume that women who attend ANC services will be more prepared for childbirth, since ANC visits are an opportunity for health care workers to inform them about danger signs and risks in pregnancy and possible complications that may arise before, during, and after birth in recent years [47]. Nevertheless, our finding that half of our study population was prepared for birth is lower than found in a study conducted in an antenatal clinic in Kenya [48].

Our study revealed that the majority of women attending the ANC at Dhulikhel Hospital had identified a health facility for childbirth and/or obstetric emergencies and had saved money for such emergencies. Similar results have been reported from other studies [14, 17]. In addition to identifying a health facility, arranging transportation is essential for reducing delays to reaching facilities when birth is imminent or in the cases of emergency due to complications during pregnancy. Only slightly over one-third, 38%, of the women had taken steps to arrange transportation, which is a low proportion compared with that found in another study in Nepal [27], and in studies conducted in the African countries, Ethiopia, Tanzania and Kenya [14, 46, 48] and India [21, 22]. The comparative lack of preparation for transportation could have been due to geographic challenges in the study area, including poor road infrastructure and limited public vehicles in most rural settings. After the earthquakes, significantly fewer women made efforts to arrange transportation (p < 0.001).

One-fifth of the women had arranged for a potential blood donor, which is notably higher than found in other studies among pregnant women in which the same type of preparation was found to be critically low [19, 24, 47], although these studies were conducted in community settings, not hospital-based ones. According to the aforementioned studies, only 3% of the pregnant women in Ethiopia, < 5% in Nepal, and 9.6% in India had identified potential blood donors in advance of childbirth. Dhulikhel Hospital organizes regular awareness campaigns in many communities through its outreach centers, which cover a wide range of public health topics, including ANC and birth preparedness. Furthermore, it is possible that the women who were attending the hospital had been informed about the importance of finding potential donors for childbirth. It is also likely that only those who had received some public health information attended the ANC.

### Other associated risk factors

Younger women were three times as likely to be unprepared for birth or complications during pregnancy compared with women who were aged thirty years or older. This result is in accordance with findings from previous studies in Tanzania [46] and India [23], and illustrates the vulnerability of young married women, living in their husband’s households, as is the cultural custom in Nepal. Furthermore, women from rural settings were 1.5 times more likely to be insufficiently prepared compared with women in urban settings in our study. This result is corroborated by two studies from Ethiopia [14, 45], where the odds of sufficient BP/CR among women who lived in with urban areas were six and two times greater, respectively. This finding may relate to the women having less access to health information through different media in rural areas, and relatively high barriers due to distance and lack of transportation compared with women in urban areas [45].

Additionally, we found a significant association between BP/CR and women’s caste and ethnicity in our study. The women belonging to the Dalit caste were three times more likely to be unprepared compared with women from upper castes. This finding is consistent with findings from an analysis of the NDHS survey done in 2011, which revealed that Dalit women utilized ANC services less than did women of other castes and ethnicities [49]. However, our finding conflicts with an Indian study that was conducted among a population of rural women who had given birth [22] and in which no significant associations were found between BP/CR and caste.

In addition, in common with other studies [13, 14, 20, 45, 46], we found that low education levels or no education were notably associated with not being prepared for childbirth or obstetric complications.

Furthermore, women who had attended less than four antenatal visits had a statistically significant higher risk of being unprepared for childbirth compared with women who had attended four or more ANC visits. Similar findings were present in Tanzania [17] and Ethiopia [45].

Women appear to be disproportionately vulnerable to violence and abuse in the aftermath of natural disasters, although research on this topic is limited. A Chinese study after the earthquake in Sichuan Province in 2008 indicated that women experienced more violence following the disaster [33]. A qualitative study with women in Australia describes both new and increased violence from male partners after the Black Saturday bushfires in 2009. [50]. In the aftermath of a natural disaster, surviving and meeting basic needs will be first priorities. Furthermore, health facilities may be destroyed or become overwhelmed, support services for women exposed to DV may be limited or non-existing, and support persons may have to deal with their own emergencies. Stress related to financial burdens on families may lead to increases in DV [2]. Due to culturally accepted gender roles, women may be tasked as the primary care takers for others in disaster situations. This combined with reduced finances and access to transportation, presents difficulties in terms of meeting their own and their families’ basic needs, and may be compounded by grief and emotional trauma following the disaster. Consequently, when the earthquakes occurred during our data collection, we decided to analyze whether the added burdens of the disaster influenced BP/CR in interaction with DV. We found an increase in the proportion of women who were unprepared for childbirth after the earthquakes compared with women who did not prepare for childbirth before the earthquakes. However, we could not detect any interference between DV, BP/CR and the earthquakes.

### Strengths and limitations of the study

This is the first comprehensive study of its kind in Nepal, where exposure to DV during pregnancy is examined as a potential risk for lack of preparedness for childbirth. This topic remains under-investigated in the world as well. The strength of the study includes its conduct among a non-selected population of pregnant women attending a hospital-based antenatal clinic. Also, the size of the study is large, allowing for multiple adjustments.

The knowledge obtained from the study makes a contribution to a previously overlooked topic in the DV research field as well, and will guide ANC providers to identify and assist women who are vulnerable to inadequate childbirth preparation due to living with DV.

We also included pregnant women under 18 years in this study (n = 11), a population that is sometimes excluded from reproductive health research due to the Helsinki Declaration, which recommends obtaining the consent of parents or guardians for all participants under 18 years. In Nepal, however, the mean age of marriage is 17.4 years [12], and the vast majority of married women are living independently from their parents. Including the insights of young women is important for improving ANC services for all women experiencing DV in Nepal. Our study therefore contributes to a fuller assessment of the prevalence of DV and BP/CR across age groups.

Another notable strength of the study is the use of a C-ACASI questionnaire, which provided an opportunity for women to respond to sensitive questions in a private, anonymous and confidential way, regardless of their literacy. We asked the women about their opinion of the technology after they had completed the questionnaire. The overwhelming majority (96%) reported that they found the method simple. Most importantly, it allowed for the inclusion of an illiterate population, and illiteracy was one of the most significant risk factors for inadequate BP/CR.

This is a cross-sectional study which does not allow for cause and effect relationships to be made between potential risks and BP/CR. The study was hospital-based, and this may have limited our potential to generalize the finding to other settings, for example to rural areas with only outreach centers. We included pregnant women between 12 weeks and 28 weeks gestation only, and women may be more prepared for childbirth or complications at a later stage in a pregnancy.

Women with hearing and vision disabilities, or whom did not speak Nepali, were excluded from the study, which is a limitation. These women may be vulnerable groups who could have given us more information on possible experiences of DV. However, as we were using C-ACASIs on a tablet computer in Nepali language, it was practically challenging to include these women. A possible bias in our findings could be related to a qualitative study that was done six months prior to this study in the same community. The previous study targeted community members generally, and no pregnant women were included. Also, BP/CR was not one of the topics specifically discussed in the study; rather, the focus of the qualitative study was on perceptions of violence against pregnant women. However, participants in the qualitative study may have discussed the topics raised in the FGDs with friends and families, and we cannot know if this may have raised general awareness and influenced pregnant women’s preparedness for childbirth.

If pregnancy outcomes were obtained from the same women after childbirth, the results could be compared during pregnancy and post-partum; this would be a valuable future study. Similarly, we did not obtain any information about women’s knowledge of obstetric danger signs. This knowledge is important for assessing BP/CR and newborn complications. However, the primary aim of the study was to evaluate if DV influenced the five BP/CR activities [3]. Likewise, data were collected based on pregnant women’s perspectives. Since men’s involvement is crucial for the realization of birth plans and complication readiness [3], innovative strategies to increase men’s awareness of obstetric danger signs and BP/CR are required to significantly improve situations for pregnant women [51]. Finally, underreporting of DV in this study is possible. In spite of the anonymity provided by the C-ACASI questionnaire, women may not have answered the questions about DV truthfully nor understood their personal experiences to be types of DV. As trust between a pregnant woman and her ANC providers is likely to increase over the course of several meetings, women might be more willing to disclose violence in their family situations at a later ANC appointment or stage in pregnancy.

## Conclusions

This study identified pregnant women attending an antenatal clinic in Nepal who had experienced DV, which affected their BP/CR. These vulnerable women may need extra care and attention in the health system in order to reduce the potentially harmful effects of violence on maternal and neonatal health, and preparations for childbirth. To gain further information about insufficient birth preparedness, a follow-up study on the outcomes of the pregnancies affected by DV is recommended. This new knowledge could inform and guide interventions by health care providers and other stakeholders.

## Supporting information

S1 Table Study questionnaire–English version. (PDF)

S2 Table Study questionnaire–Nepali version. (PDF)
