# Supplementary material for: Exposure to domestic violence influences pregnant women’s preparedness for childbirth in Nepal: A cross-sectional study
Source: PLoS One. 2018 Jul 26;13(7):e0200234. doi: 10.1371/journal.pone.0200234 (PMC6061992; doi:10.1371/journal.pone.0200234)
Supplement: S1 Table — (PDF) [file pone.0200234.s001.pdf]

Study questionnaire

Pregnancy and women's reproductive health in Nepal

Code number:
Date of interview:
Last menstrual period:

**Section A: Obstetric History**			
**Q #**	**Question**	**Code**	**Go to Q**
1	Is this your first pregnancy?	Yes………………..1 → No…………………2	6
2	How many children have you given birth to?	None……………. 1 → 1……………………2 2……………………3 3……………………4 4 or more………….5	5
3	How many live boys do you have?	None……………. 1 1……………………2 2……………………3 3……………………4 4 or more………….5	
4	How many live girls do you have?	None……………. 1 1……………………2 2……………………3 3……………………4 4 or more………….5	
5	Have you given birth to any baby who died during or shortly after birth?	None……………. 1 1……………………2 2……………………3 3……………………4 4 or more………….5	
6	Have you had any treatment for infertility in relation to this present pregnancy?	Yes………………..1 No…………………2	
7	Did your family pressure you into this pregnancy?	Yes………………..1 No…………………2	
8	How many antenatal check-ups have you had during this pregnancy?	1……………………1 2……………………2 3……………………3 4 or more…………..4	

9	Have you ever had any spontaneous abortion/s?	None……………….1<br>Less than 3………..2<br>3 or more…………..3	
10	Have you ever had induced abortion/s?	None……………….1<br>Less than 3………..2<br>3 or more…………..3	
**Section B:Socio-demographic History**			
11	How old are you (in years)?	Age in years…… ☐☐	
12	Where do you live?	Urban……………..1<br>Rural……………….3	
13	What level of education/literacy have you completed?	Cannot read & write……………..1<br>Can read & write some…………..2<br>Completed up to grade 5………..3<br>Completed up to grade 10……..4<br>Completed up to grade 12 or more..5	
14	Do you have any independent income of your own?<br><br>14.1. If yes, what is your personal monthly income?	Yes………………..1 →<br>No…………………2 →<br><br>Less than Rs 8,000………………..1<br>Between Rs 8,000 and Rs16,000...2<br>More than Rs 16,000……………3	14.1<br>16
15	Do you need permission from anyone from your family to use your personal income?	Yes……………………….1<br>No………………………..2	
16	Are you married?	Yes………………..1 →<br>No…………………2	19
17	Are you a widow?	Yes………………..1 →<br>No…………………2	19
18	Are you divorced?	Yes………………..1 →<br>No…………………2	20
19	What type of family do you live in?	Nuclear family………..1<br>Joint family…………,,.2<br>Extended joint family..3	
20	Do you have a husband or partner?<br><br>20.1. If yes, do you live with your husband/partner	Yes………………..1<br>No…………………2 →<br><br>Yes………………..1<br>No…………………2 →	29<br><br>29
21	How old is your husband/partner?	Age in years…… ☐☐	
22	How long have you been living with your husband/partner?	Less than 1 year…………………1<br>More than1 year to less than 5 years...2	

		Less than 5 years to less than 10 years……………………………..3<br>10 years or more………………..4	
23	What was your age when you got married?	Age in years…… ☐☐	
24	What was your husband's age when he got married to you?	Age in years…… ☐☐	
25	Is this your first marriage?	Yes………………..1<br>No…………………2	
26	Are you your husband's only wife?	Yes………………..1<br>No…………………2	
27	What level of education/literacy has your husband/partner completed?	Cannot read & write…………….1<br>Can read & write some…………..2<br>Completed up to grade 5………..3<br>Completed up to grade 10……..4<br>Completed up to grade 12 or more.5	
28	Do you know your husband's/partner's income?<br><br>28.1. If yes, what is your husband's/partner's monthly income?	Yes………………..1<br>No…………………2 →<br><br>Less than Rs 8,000………………….1<br>Between Rs 8,000 and Rs 16,000...2<br>More than Rs.16,000…………….3	29

Section C: Violence Questions

Research has shown that some life experiences might have negative effects on the health of a pregnant mother and her baby. This section will ask you some sensitive questions. Your participation is voluntary and you may withdraw from this study without giving any explanation. Sharing your experience may contribute to improving the quality of antenatal care in Nepal.

29	Have you ever been asked about family violence by any health care provider?	Yes………………………..1<br>No………………………..2	
30	Are you afraid of anyone within your family?<br><br>30.1. If yes, whom?<br><br>30.2. How often have you been afraid within the last month?	Yes………………………..1<br>No………………………..2 →<br><br>Husband…………………..,,,1<br>Ex-husband…………………..2<br>Boyfriend……………………..3<br>Mother in law………………..4<br>Father in law…………………5<br>Other family members…..6<br><br>Seldom…………………………1<br>Sometimes……………………2<br>Often……………………………3<br>Most of the time…………..4	31

31	Have you ever been emotionally or physically abused by someone important in the family to you?<br><br>31.1. If yes, whom?<br><br><br><br><br><br><br>31.2. How many times have you ever been emotionally or physically abused by someone important in the family to you?	Yes……………………….1<br>No……………………….2<br><br><br>Husband………………………..1<br>Ex-husband……………….......2<br>Boyfriend……………….........3<br>Mother in law……………......4<br>Father in law……………….....5<br>Other family members…….6<br><br>Once or twice……………………………...1<br>Several times (3-5 times)……………….2<br>Many times (more than 5 times)……3	→ 32
32	Within the last year, have you been hit, slapped, kicked or otherwise physically hurt by someone in the family?<br><br>32.1. If yes, by whom?<br><br><br><br><br><br><br><br>32.2. How many times have you been hit, slapped, kicked or otherwise physically hurt within the last year by someone in the family?	Yes……………………….1<br>No……………………….2<br><br><br>Husband………………………..1<br>Ex-husband…………….........2<br>Boyfriend……………….…........3<br>Mother in law…………….......4<br>Father in law…………….,,,,,,5<br>Other family members……..6<br><br>Once or twice………………………...1<br>Several times (3-5 times)…………….2<br>Many times (more than 5 times)…3	→ 33
33	Since you've been pregnant, have you been hit, slapped, kicked or otherwise physically hurt by someone in the family?<br><br>33.1. If yes, by whom?<br><br><br><br><br><br><br>33.2. How many times have you been hit, slapped, kicked or otherwise physically hurt by someone in the family since you have been pregnant?	Yes……………………….1<br>No……………………….2<br><br><br>Husband…………………..1<br>Ex-husband…………….......2<br>Boyfriend………………......3<br>Mother in law…………….....4<br>Father in law……………….5<br>Other family members……..6<br><br>Once or twice………………..1<br>Several times (3-5 times)……2<br>Many times (more than 5 times)………3	→ 34

34	Within the last year has anyone in the family forced you to have sexual activities?<br><br>34.1. If yes, whom?<br><br><br><br><br><br><br><br>34.2. How many times have you been forced to have sexual activities by someone in the family within last year?	Yes…………………………1<br>No…………………………2 →<br><br><br>Husband……………………1<br>Ex-husband…………………..2<br>Boyfriend…………………..3<br>Mother in law………………..4<br>Father in law………………..5<br>Other family members……..6<br><br>Once or twice………………..1<br>Several times (3-5 times)……2<br>Many times (more than 5 times)…………3	35
If you answered "yes" to question 30, 31, 32, 33 and/or 34 above, then please also answer question 35 below:			
35	Have you ever told any of your health care providers about your experience(s) of violence?	Yes………………..1<br>No…………………2	
**Section D: Attitudes about Domestic Violence -** People have different ideas about families and what is acceptable behaviour for men and women in the home.			
36	In your opinion, does a man have a good reason to hit his wife if:<br>36.1. She does not complete her household work to his satisfaction<br><br><br>36.2. She disobeys him<br><br><br><br>36.3. She refuses to have sexual relations with him<br><br><br>36.4. She asks him whether he has other girlfriends<br><br><br>36.5. He suspects or finds out that she is Unfaithful	 <br>Yes……………………………1<br>No……………………………..2<br>Don't know…………………8<br><br>Yes……………………………..1<br>No……………………………..2<br>Don't know……………………8<br><br>Yes……………………………..1<br>No……………………………..2<br>Don't know……………………8<br><br>Yes……………………………..1<br>No……………………………..2<br>Don't know……………………8<br><br>Yes……………………………..1<br>No……………………………..2<br>Don't know……………………8	
**Section E: Birth Preparedness and Complication Readiness**			

37	(Only to those who have experienced a pregnancy before)<br>Did you have any complications during any of your previous pregnancies, either during the delivery or directly after birth?	Yes……………………………1<br>No………………………………2<br>Not applicable..............................3		 →39<br>→39
38	If yes, did you have:<br>38.1. High blood pressure?<br><br>38.2. Bleeding (vaginal)<br><br>38.3. Prolonged labour (more than 12 hours)<br><br>38.4. Infection<br><br>38.5. Cesarean section	 Yes……………………………1<br>No………………………………2<br><br>Yes……………………………1<br>No………………………………2<br><br>Yes……………………………1<br>No………………………………2<br><br>Yes……………………………1<br>No………………………………2<br><br>Yes……………………………1<br>No………………………………2		
39	Which arrangements (steps), if any, have you or your family made for the birth of this child?	Yes	No	
	39.1. Identified a skilled birth attendant for the delivery.	1	2	
	39.2. Identified a health facility.	1	2	
	39.3. Arranged for transport for the delivery.	1	2	
	39.4. Identified a potential blood donor.	1	2	
	39.5. Saved money for a possible emergency.	1	2	
40	Do you know if there is any incentive to receive for giving birth at a health facility?<br><br>40.1. If yes, can you use the money yourself?	Yes………………………..1<br>No…………………………2<br><br>Yes………………………..1<br>No…………………………2		 →41
41	Did someone accompany you here for this antenatal check up?<br><br>41.1. If yes, who brought you here?	Yes………………………..1<br>No…………………………2<br><br>Husband/partner…………..1<br>Mother/father in law……….2<br>Your child/ren……………..3<br>Neighbour/friend………….4<br>Others.................................5		 →42

Section F: Emotional Wellbeing (HSCL-5):						
42	Within the last 14 days have you been:	Not at all	A little	Quite a bit	Extremely	
	42.1. Feeling fearful.	1	2	3	4	
	42.2. Experienced nervousness or shakiness	1	2	3	4	
	42.3. Feeling hopeless about the future	1	2	3	4	
	42.4. Feeling blue	1	2	3	4	
	42.5. Worrying too much about things	1	2	3	4	

Thank you for your participation!
