# Supplementary material for: Exposure to domestic violence influences pregnant women’s preparedness for childbirth in Nepal: A cross-sectional study
Source: PLoS One. 2018 Jul 26;13(7):e0200234. doi: 10.1371/journal.pone.0200234 (PMC6061992; doi:10.1371/journal.pone.0200234)
Supplement: S2 Table — (PDF) [file pone.0200234.s002.pdf]

अनुसूची–३

गर्वास्था र महीलाको प्रजनन स्वास्थ्य: अध्ययन प्रश्नावली

कोड नम्बर :
अन्तर्वार्ता मिति :
अन्तिम महिनावारी :

भाग 'क' : प्रसूति विवरण			
प्र. #	प्रश्न	कोड	प्रश्नमा जानुहोस्
१.	के यो तपाईंको पहिलो गर्भ हो ?	हो....................... १<br>होइन.................... २	→ ६
२.	तपाईंले कतिजना बच्चा जन्माउनु भयो ?	छैन.........................१<br>१............................२<br>२............................३<br>३............................४<br>४ वा बढी.................५	→ ५
३.	तपाईंको कतिजना छोराहरु जीवित छन् ?	छैन.........................१<br>१............................२<br>२............................३<br>३............................४<br>४ वा बढी.................५	
४.	तपाईंको कतिजना छोरीहरू जीवित छन् ?	छैन.........................१<br>१............................२<br>२............................३<br>३............................४<br>४ वा बढी.................५	
५.	के तपाईंको कुनै बच्चा जन्मिने क्रममा या जन्मिने बित्तिकै मृत्यु भएको थियो ?	थिएँ.........................१<br>थिएन.......................१	
६.	के तपाईंले अहिलेको गर्भमा रहेको बच्चाको सम्बन्धमा कुनै बाँझोपनको उपचार गर्नुभएको थियो ?	थिएँ.........................१<br>थिएन.......................२	
७.	तपाईँको ईच्छाविपरित तपाईँलाई परिवारले गर्भवती हुनलाई बाध्य बनाएको हो ?	हो..................... .....१<br>होइन........................२	

८.	यो गर्भावस्थामा तपाईंले कतिपटक जाँच गराउनुभयो ?	१............................१<br>२............................२<br>३............................३<br>४ वा बढी.................४	
९.	तपाईंको कुनै गर्भ आफैं खेर गएको छ ?	छैन.........................१<br>३ भन्दा कम..............२<br>३ भन्दा बढी..............३	
१०.	के तपाईंले कहिल्यै गर्भपतन गराउनु भएको छ ?	छैन.........................१<br>३ भन्दा कम..............२<br>३ भन्दा बढी..............३	
**भाग 'ख' : सामाजिक जनसाङ्ख्यिक विवरण**			
११.	तपाईं कति वर्षको हुनुभयो ?	वर्षमा उमेर........ ☐☐	
१२.	तपाईं कहाँ बस्नु हुन्छ ?	शहर.......................१<br>गाउँ.......................२	
१३	तपाईंले कुन तहसम्मको पढाइ पूरा गर्नु भएको छ ?	पढ्न र लेख्न सक्दिन..........१<br>केही मात्रामा पढ्न र लेख्न सक्छु.............................२<br>कक्षा ५ सम्म...................३<br>कक्षा १०सम्म...................४<br>कक्षा१२ वा त्यो भन्दा माथि....५	
१४.	के तपाईंको आफ्नो कमाइ छ ?<br><br>१४.१ यदि छ भने, तपाइँको मासिक आम्दानी कति छ ?	छ..........................१<br>छैन.........................२<br><br>रु ८००० भन्दा मुनी ....१<br>रु ८००० देखि रु १६००० सम्म .....२<br>रु १६००० भन्दा माथि ३	→ १४.१<br>→ १६
१५.	के तपाईंलाई आफ्नो कमाई स्वतन्त्ररुपले खर्च गर्न परिवारका अनुमति लिनुपर्छ ?	पर्छ...........................१<br>पर्दैन.........................२	
१६.	के तपाईं विवाहित हुनुहुन्छ ?	छ..............................१<br>छैन २	→१९
१७.	के तपाईं विधवा हुनुहुन्छ ?	हो.........................१<br><br>हैन.........................२	→१९
१८.	के तपाईंको सम्बन्ध विच्छेद भएको हो ?	हो.........................१	→२०

		होइन......................२	
१९.	तपाईं कस्तो प्रकारको परिवारमा बस्नुहुन्छ ?	एकल.......................१<br>संयुक्त......................२<br>वृहत........................३	
२०.	के तपाईंको श्रीमान्/प्रेमी हुनुहुन्छ ?<br><br>२०.१ यदि हुनुहुन्छ भने के तपाईं आफ्नो श्रीमान् / प्रेमीसँग बस्नुहुन्छ ?	छ.........................१<br>छैन........... ....२ →<br><br>बस्छु......................१<br>बस्दिन....................२ →	२९<br><br><br>२९
२१.	तपाईंको श्रीमान्/प्रेमी कति वर्षको हुनुभयो ?	वर्षमा उमेर .... ☐☐	
२२.	तपाईं आफ्नो श्रीमान्/प्रेमीसँग बस्न ुभएको कति भयो ?	१ वर्षभन्दाकम.....................१<br>१ वर्षभन्दा बढी–५ वर्ष भन्दा कम..................................२<br>५ वर्षभन्दा बढी–१० वर्षभन्दा कम...................................३<br>१० वर्ष वा सोभन्दा बढी.................................४	
२३.	तपाईंको विवाह हुँदा तपाईँ कति वर्षको हुनुहुन्थ्यो ?	वर्षमा उमेर............. ☐☐	
२४.	तपाईंको विवाह हुँदा तपाईंको श्रीमान् कति वर्षको हुनुहुन्थ्यो ?	वषमा उमेर............. ☐☐	
२५.	के यो तपाईंको पहिलो विवाह हो ?	हो.................................१<br>होइन..............................२	
२६.	के तपाईंको श्रीमान्को तपाईं एक्ली श्रीमति हुनुहुन्छ ?	हो.................................१<br>होइन..............................२	
२७.	तपाईंको श्रीमान्/प्रेमीले कुन तहसम्मको पढाइ पूरा गर्नु भएको छ ?	पढ्न र लेख्न सक्नु हुदैन.....१<br>केही मात्रामा पढ्न र लेख्न सक्नुहुन्छ.........................२<br>कक्षा ५ सम्म ....३<br>कक्षा १०सम्म.................४<br>कक्षा १२ वा त्यो भन्दा माथि...५	

२८.	तपाईंलाई तपाईंको श्रीमान्/प्रेमीको आम्दानी बारे थाहा छ ?<br><br>२८.१ यदि थाहा छ भने तपाईंको श्रीमान/प्रेमीको मासिक आम्दानी कति छ ?	थाहा छ............................१<br>थाहा छैन.........................२<br><br>रु ८००० भन्दा मुनी ....१<br>रु ८००० देखि रु १६००० सम्म .....२<br>रु १६००० भन्दा माथि ३	→ २९

भाग 'ग' : हिँसा सम्बन्धी प्रश्नहरू

अनुसन्धानले देखाए अनुसार जीवनमा भोगेका अनुभवहरुले गर्भावस्थामा आमा तथा बच्चाको स्वास्थ्यमा नकरात्मक असर पारेको हुनसक्छ । यस भागमा संवेदनशिल प्रश्नहरु हुनसक्छ । तपाइको सहभागिता स्वेच्छिक हुनेछ र तपाई कुनै कारण नजनाइकन पनि यो अध्ययनलाई छोड्न सक्नुहुनेछ। तपाइले बताउनु भएको अनुभवको आधारमा हिंसामा परेका महिलाको गर्भावस्थाको जाँच तथा सेवाको गुणस्तरमा सुधार ल्याउनमा मद्दत पुग्न सक्छ ।

२९.	के तपाईंलाई कुनै स्वास्थ्य सेवा प्रदायकले घरेलु हिँसा सम्बन्धी सोधेको छ ?	छ.........................१<br>छैन.......................२	
३०.	के तपाईंलाई आफ्नो परिवारको कुनै सदस्यसँग डर लाग्छ ?<br><br>३०.१ यदि डर लाग्छ भने, को सँग ?<br><br>३०.२ गएको महिनाभित्रमा तपाईं कतिपटक डराउनु भयो ?	लाग्छ.........................१<br>लाग्दैन.......................२<br><br>श्रीमान्.............................१<br>पूर्व श्रीमान्.......................२<br>प्रेमी................................३<br>सासु...............................४<br>ससुरा..............................५<br>परिवारको अन्य सदस्य.........६<br><br>विरलै..............................१<br>कहिलेकाँही......................२<br>धेरै जसो.........................३<br>सधैंजसो.........................४	→ ३१
३१	के तपाईं कहिल्यै भावानात्मक वा शारीरिकरूपले परिवारको महत्वपूण सदस्यबाट दुर्व्यवहारमा पर्नु भएको छ ?	छ.........................१<br>छैन.......................२	→ ३२

	३१.१ यदि छ भने कोबाट ?	श्रीमान्...............................१<br>पूर्व श्रीमान्.........................२<br>प्रेमी.................................३<br>सासू................................४<br>ससुरा...............................५<br>परिवारको अन्य सदस्यहरू......६	
	३१.२ कतिपटक तपाईं भावानात्मक वा शारीरिकरूपले परिवारको महत्वपूर्ण सदस्यबाट दुर्व्यवहारमा पर्नु भएको छ ?	एक वा दुई पटक.......................१<br>केही पटक (३–५ पटक)...............२<br>धेरै पटक (५ पटक भन्दा माथि)......३	
३२.	गत वर्षभित्रमा के तपाईंलाई परिवारको महत्वपूर्ण सदस्यले कुटेको, थप्पड हानेको, लातले हानेको वा अन्य शारीरिकरूपमा चोट पुऱ्याएको छ ?	छ.........................१<br>छैन........................२	→३३
	३२.१ यदि छ भने कसले ?	श्रीमान्.............................१<br>पूर्व श्रीमान्........................२<br>प्रेमी.................................३<br>सासु................................४<br>ससुरा...............................५<br>परिवारको अन्य सदस्य..........६	
	३२.२ गत वर्षभित्र कतिपटक तपाईंलाई परिवारको महत्वपूर्ण सदस्यले कुटेको, थप्पड हानेको, लातले हानेको वा अन्य शारीरिकरूपमा चोट पुऱ्याएको छ ?	एक वा दुई पटक......................१<br>केही पटक (३–५ पटक)..............२<br>धेरै पटक (५ पटक भन्दा माथि).....३	
३३.	तपाईँ गर्भवती भएदेखि तपाईंलाई परिवारको कुनै सदस्यले कुटेको, थप्पड हानेको, लातले हानेको वा अन्य शारीरिकरूपमा चोट पुऱ्याएको छ ?	छ..१<br>छैन.......................................२	→३४
	३३.१ यदि छ भने कसले ?	श्रीमान्..................................१<br>पूर्व श्रीमान्.............................२<br>प्रेमी.....................................३<br>सासू.....................................४	

	 <br><br>३३.२ तपाइँ गर्भवती भएदेखि तपाईलाई परिवारको कुनै सदस्यले कतिपटक कुटेको, थप्पड हानेको, लातले हानेको वा अन्य शारीरिकरूपमा चोट पुऱ्याएको छ ?	ससुरा.................................५<br>परिवारको अन्य सदस्य..............६<br><br>एक वा दुई पटक......................१<br>केही पटक (३–५ पटक)..............२<br>धेरै पटक (५ पटक भन्दा माथि)......३	
३४.	गएको वर्षभित्रमा तपाइँलाई परिवारमा कसैले यौनक्रिया कलापको लागी बाध्य बनाएको थियो ?<br><br>३४.१ यदि थियो भने कसले ?<br><br><br><br><br><br><br>३४.२ गत वर्षभित्रमा परिवारको कुनै सदस्यले तपाइँलाई कतिपटक यौनक्रियाकलापको लागि बाध्य बनाएको थियो ?	थियो............................१<br>थिएन...........................२ →<br><br><br>श्रीमान्............................१<br>पूर्व श्रीमान्........................२<br>प्रेमी................................३<br>सासू...............................४<br>ससुरा..............................५<br>परिवारको अन्य सदस्य.........६<br><br>एक वा दुई पटक.....................१<br>केही पटक (३–५ पटक).............२<br>धेरै पटक (५ पटक भन्दा माथि).....३	 ३५

यदि तपाइले प्रश्नहरु ३०,३१,३२,३३ र/वा ३४ माथि हो जवाफ दिनुभएमा कृपया तलका ३५ को पनि जवाफ दिनुहोला :

३५.	के तपाईंले कुनै स्वास्थ्य सेवा प्रदायकलाई आफ्नो घरेलु हिंसा सम्बन्धी अनुभवहरू भन्नु भएकोछ ?	छ...१<br>छैन.......................................२	

भाग 'घ' : घरायसी हिँसा सम्बन्धी प्रवृत्ति : मानिसहरूको परिवार सम्बन्धी साथै घरमा पुरुष तथा महिलाहरूले गरिने स्वीकारयोग्य व्यहार सम्बन्धी पनि भिन्नभिन्न धारणा हुन्छ ।

३६.	तपाईंको विचारमा के एक श्रीमान्ले आफ्नो श्रीमतीलाई पिट्न उचित छ, यदि :<br><br>३६.१ श्रीमतीले श्रीमानको संतोष हुने गरी घरको काम गर्दिनन् भने ।	 <br>छ...............................१<br>छैन.............................२<br>थाहा छैन......................३	

	३६.२ श्रीमतीले श्रीमान्को अवज्ञा गर्छिन् भने ।	छ...............................१<br>छैन.............................२<br>थाहा छैन......................३	
	३६.३ श्रीमतीले श्रीमान्सँग यौन सम्बन्ध राख्न अस्वीकार गर्छिन् भने ।	छ...............................१<br>छैन.............................२<br>थाहा छैन......................३	
	३६.४ श्रीमतीले श्रीमान्लाई प्रेमीका छन् की छैनन् भनेर प्रश्न गर्छिन् भने ।	छ...............................१<br>छैन.............................२<br>थाहा छैन......................३	
	३६.५ श्रीमान्ले आफ्नो श्रीमती परपुरुषसँग सम्बन्ध जोडेको शंका गर्छन भने ।	छ...............................१<br>छैन.............................२<br>थाहा छैन......................३	
**भाग 'ङ' : प्रसव पूर्व तयारी :**			
३७.	(ती महिलालाई मात्र जो पहिले गर्भवती भैसकेकीछिन्)<br>के तपाईंले तपाईंको पछिल्लो गर्भावस्थामा प्रसवको बेला वा जन्मको लगतै कुनै समस्या भोग्नु परेको थियो ?	थियो...................१<br>थिएन..................२ →<br>लागु हुँदैने............३ →	 ३९<br>३९
३८.	यदि समस्या थियो भने के :<br>३८.१ उच्च रक्तचाप थियो ?	थियो...........................१<br>थिएन..........................२	
	३८.२ रक्तस्राव (योनिबाट) थियो ?	थियो...........................१<br>थिएन..........................२	
	३८.३ लामो समयसम्म प्रसव व्यथा (१२ घण्टा भन्दा बढी) लागेको थियो ?	थियो...........................१<br>थिएन..........................२	
	३८.४ संक्रमण भएको थियो ?	थियो...........................१<br>थिएन..........................२	

	३८.५ पेट चिरेर (सीजेरियन सेक्सन) बच्चा निकालेको थियो ?	थियो..........................१<br>थिएन..........................२		
३९.	तपाईं र तपाईंको परिवारले बच्चा जन्मनको लागि के के तयारी (व्यवस्था) गर्नुभयो ?	गरियो	गरिएन	
	३९.१ तालिमप्राप्त प्रदायकको पहिचान गर्नु भयो ?	१	२	
	३९.२ प्रसूति गराउने स्वास्थ्यसेवा केन्द्र पत्ता लगाउनु भयो ?	१	२	
	३९.३ प्रसवको समयमा यातायातको व्यवस्था गर्नु भयो ?	१	२	
	३९.४ रगत चाहियो भने दिने व्यक्तिको पहिचान गर्नु भयो ?	१	२	
	३९.५ हुनसक्ने आपतकालीन अवस्थाको लागि पैसा जम्मा गर्नु भयो ?	१	२	
४० .	तपाईंले स्वास्थ्य केन्द्रमा बच्चा जन्माउनु भयो भने प्रोत्सान स्वरूप केही पैसा पाइन्छ भन्ने थाहा छ कि छैन ?	थाहा छ..............१<br>थाहा छैन............२ →		४१
	४०.१ यदि थाहा छ भने के तपाईं आफ्नो लागि त्यो पैसा खर्च गर्न सक्नुहुन्छ ?	सक्छु...............१<br>सक्दिन............२		
४१.	यो गभवती जाँच गराउनको लागि के तपाईंसँग कोही आउनुभएको छ ?	छ..................१ →<br>छैनन्..............२ →		४२
	४१.१ यदि आउनुभयो भने को आउनुभयो ?	श्रीमान्/प्रेमी..................१<br>सासू/ससूरा...................२<br>तपाईंको बच्चा................३<br>छिमेकी/साथी.................४<br>अन्य.............................५		

भाग 'च' : भावनात्मक सुअवस्था

		कहिल्यै भएन	कहिले काही भयो	धेरै जसो भयो	एकदम धेरै पटक भयो
४२ .	गएेको १४ दिनभित्रमा के तपाईर्ले :				
	४२.१ डराएको महसुस गर्नु भयो ?	१	२	३	४
	४२.२ अत्तालिएको अथवा कामेको जस्तो भयो ?	१	२	३	४
	४२.३ भविष्य बारे निराशा हुनुभयो ?	१	२	३	४
		१	२	३	४

	४२.४ उदास/दुःखी भएको महसुस गर्नु भयो ?<br>४२.५ केही कुरा बारे अति नै चिन्तित हुनुभयो ?	१	२	३	४	

तपाईंको सहभागिताको लागि धन्यवाद !
